# Cross-Neutralization between Human and African Bat Mumps Viruses

**DOI:** 10.3201/eid2204.151116

**Published:** 2016-04

**Authors:** Hiroshi Katoh, Toru Kubota, Toshiaki Ihara, Ken Maeda, Makoto Takeda, Minoru Kidokoro

**Affiliations:** National Institute of Infectious Diseases, Tokyo, Japan (H. Katoh, T. Kubota, M. Takeda, M. Kidokoro);; Mie Hospital, Mie, Japan (T. Ihara);; Yamaguchi University, Yamaguchi, Japan (K. Maeda)

**Keywords:** bat virus, Chimeric virus, mumps virus, African bat mumps virus, neutralization, viruses

## Abstract

Recently, a new paramyxovirus closely related to human mumps virus (MuV) was detected in bats. We generated recombinant MuVs carrying either or both of the fusion and hemagglutinin-neuraminidase bat virus glycoproteins. These viruses showed replication kinetics similar to human MuV in cultured cells and were neutralized efficiently by serum from healthy humans.

Many batborne paramyxoviruses closely related to mammalian paramyxoviruses recently have been identified, suggesting a possible risk for transmission of batborne paramyxoviruses to humans (*1*). Although no infectious virus has been isolated, the genome of a new paramyxovirus detected in an epauletted fruit bat (*Epomophorus* sp.) in the Democratic Republic of the Congo was closely related to the mumps virus (MuV, genus *Rubulavirus*) (*2*).

Mumps is typically characterized by inflammation of the parotid glands but also can be accompanied by orchitis, aseptic meningitis, pancreatitis, and deafness (*3*). Mumps vaccines have been used worldwide for >20 years. MuV is serologically monotypic (*4*). The fusion (F) and hemagglutinin-neuraminidase (HN) proteins, but not the small hydrophobic (SH) membrane protein, are the major targets of neutralizing (NT) antibodies (*5*,*6*).

By using expression plasmids, in 2015, Kruger et al. determined that the envelope proteins of the new paramyxovirus African bat MuV (ABMuV) were serologically and functionally related to those of MuV (*7*). We generated infectious recombinant MuVs (rMuVs) carrying either or both of the F and HN glycoproteins of ABMuV to analyze their functions and serologic cross-reactivities in the context of virus infection.

## The Study

**Figure 1:**
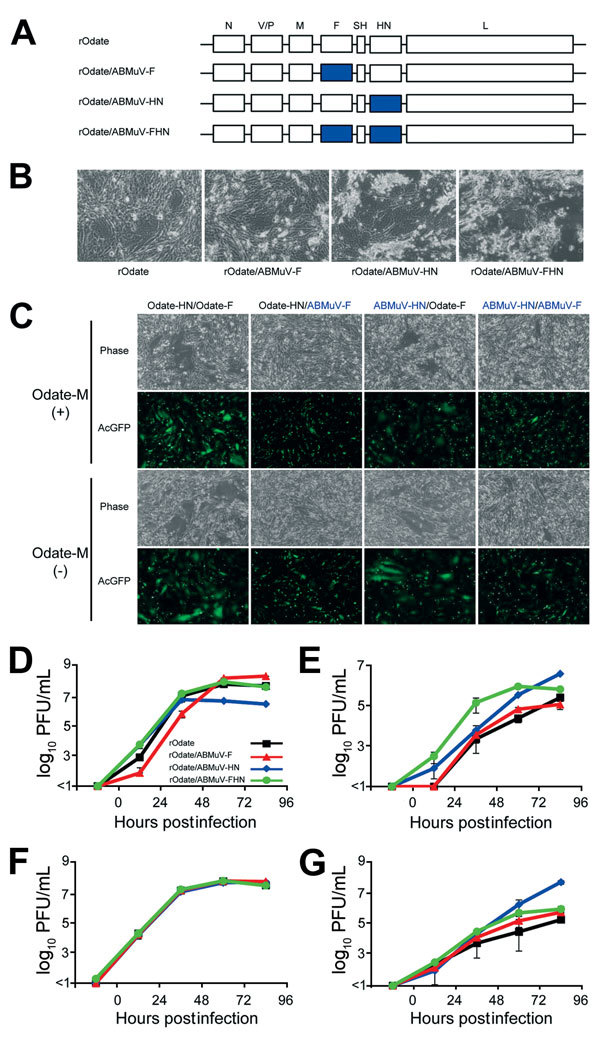
Construction of rMuVs expressing the ABMuV envelope proteins for study of their functions. A) Genome structures of the rMuVs. The 7 boxes indicate the N, V/P, M, F, SH, HN, and L genes of MuV. The blue boxes indicate the genes derived from ABMuV. B) Cytopathic effect of rMuV infection of the Vero cells followed by incubation for 48 hr. C) BHK cells were transfected with expression plasmids of the HN and F proteins (pCAGGS-Odate-HN or -ABMuV-HN and pCAGGS-Odate-F or -ABMuV-F). They were also cotransfected with expression plasmids of the M protein (pCAGGS-Odate-M) and AcGFP (pAcGFP-C1, Clontech). At 48 h posttransfection, the cells were observed under a phase-contrast and a fluorescence microscope. D–G) Growth kinetics of the rMuVs in Vero (African green monkey) (D), A549 (human) (E), THP-1 (human) (F) and FBKT1 (Ryukyu fruit bat) (G) cells. Each cell line was infected with the rMuVs at a multiplicity of infection of 0.01. At the indicated times postinfection, the culture supernatants were collected, and the infectious titers were determined by plaque assay. ABMuV, African bat mumps virus; F, fusion; HN, hemagglutinin-neuraminidase; L, large; M, matrix; N, nucleocapsid; rMuV, recombinant mumps virus; SH, small hydrophobic; P, phosphoprotein; V, historically considered the fifth viral protein. Error bars indicate SD.

A full-length genomic cDNA of the MuV Odate strain (pMuV-Odate) (*8*) was constructed, and the open reading frames of the F and HN genes were exchanged individually or together with those of ABMuV, which were obtained by an artificial composition (GenBank accession no. HQ660095) (pMuV-Odate/ABMuV-F, /ABMuV-HN, and /ABMuV-FHN) (Figure 1, panel A). All infectious viruses were rescued by transfecting the plasmids pMuV-Odate, /ABMuV-F, /ABMuV-HN, and /ABMuV-FHN, along with helper plasmids, into BHK/T7–9 cells. Rescued viruses were propagated in Vero cells.

First, we examined syncytium formation in the Vero cells infected with all of the rMuVs (Figure 1, panel B). Although rOdate/ABMuV-F produced a cytopathic effect similar to that of rOdate, larger syncytia and severe cell detachment were observed in the cells infected with rOdate/ABMuV-HN and -FHN. To understand the basis for the enhanced syncytium formation by rMuVs carrying the ABMuV HN protein, we expressed the F and HN proteins of the Odate and ABMuV strains using expression plasmids and examined for syncytium formation. Expression of the ABMuV HN protein did not enhance syncytium formation when used in expression plasmids (Figure 1, panel C). These data were consistent with a previous study (*7*). The matrix protein was expressed together with the HN and F proteins because it can modulate syncytium formation. However, it did not affect the fusion activity. Therefore, the findings with infectious viruses appeared to differ from those obtained with expression plasmids (*7*).

All rMuVs were propagated efficiently in Vero cells (Figure 1, panel D). Although at 24 and 48 h postinfection the titers of rOdate/ABMuV-F were lower than those of the other 3 viruses, they later increased to ≈10^8^ PFU/mL, which was comparable to the titers of rOdate and rOdate/ABMuV-FHN. On the other hand, the peak titer of rOdate/ABMuV-HN was as low as 5 × 10^6^ PFU/mL. To determine whether the envelope proteins affect the cell tropisms of MuV in vitro*,* we evaluated the viral growth in human- and bat-derived cell lines. Human lung epithelial A549 and human monocytic THP-1 cells were used because epithelial cells and monocytes are the primary targets of MuV in vivo (*9*). In A549 cells, rOdate/ABMuV-HN showed the highest titer by up to >10^6^ PFU/mL at 96 h postinfection (Figure 1, panel E). The other 3 rMuVs also replicated well in A549 cells up to ≈10^5^ PFU/mL, with rOdate/ABMuV-FHN showing much faster kinetics than the others. All 4 viruses grew to similar titers of up to 10^7^ PFU/mL in THP-1 cells (Figure 1, panel F). Growth was also efficient in the fruit bat–derived FBKT1 cells, although the peak titers of rOdate were lowest (Figure 1, panel G). Collectively, these findings using culture cells suggested that the envelope proteins are not a critical determinant of host specificity between ABMuV and MuV.

**Figure 2:**
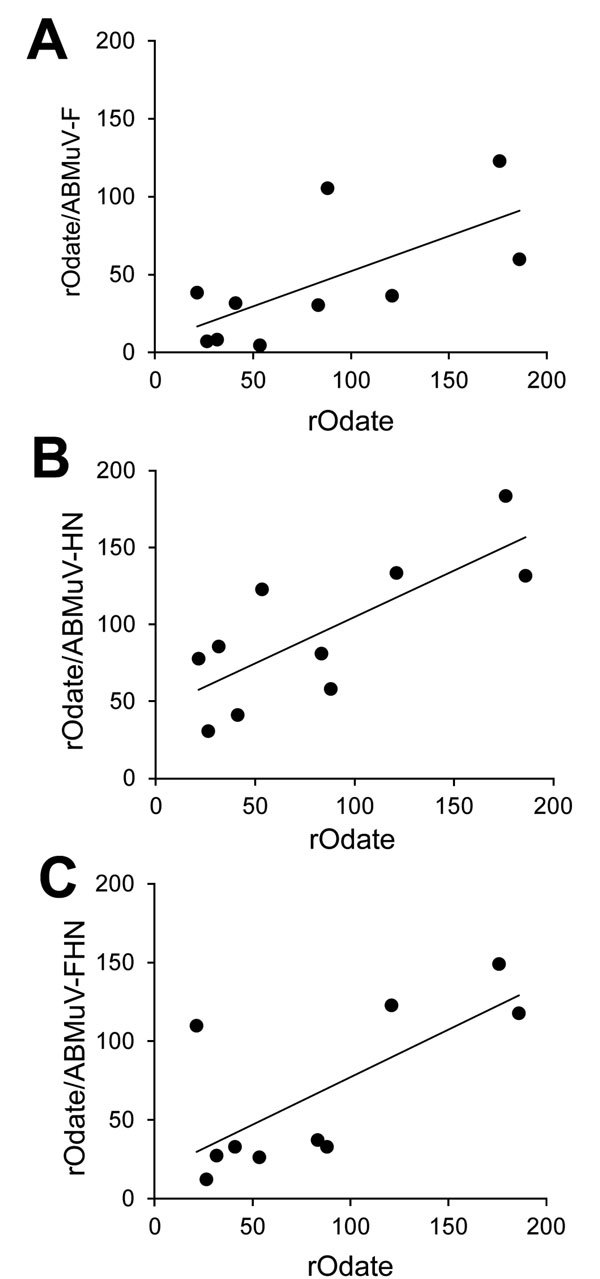
Comparison of the NT titer of rOdate versus rOdate/ABMuV-F (A), -HN (B), and -FHN (C) in a study of serologic cross-reactivities. r and p values, calculated by using the Pearson product-moment correlation, are as follows: (A) r = 0.67, p<0.05; (B) r = 0.77, p<0.01; (C) r = 0.71, p<0.05. ABMuV, African bat mumps virus; F, fusion; HN, hemagglutinin-neuraminidase; NT, neutralizing.

We conducted NT assays and ELISA using human serum obtained from 12 healthy adults (18–58 years of age) under approval by the Ethical Committees of National Institute of Infectious Diseases. Ten of 12 serum specimens (nos. 1–10) were seropositive or indeterminate (titer >2^1^) and neutralized rOdate (NT titer >4-fold) (Table). The MuV-NT serum samples showed cross-neutralization between rOdate and 3 chimeric MuVs (Table). Correlations of the NT titers were significant among rOdate and rOdate/ABMuV-F, -HN and –FHN of 0.67 (p<0.05), 0.77 (p<0.01), and 0.71 (p<0.05), respectively, by Pearson product-moment correlation (Figure 2). In addition, serum from a rabbit vaccinated with a genotype B mumps vaccine strain also neutralized the rMuVs carrying the ABMuV envelope proteins (data not shown). All data demonstrated that MuV and ABMuV were serologically cross-reactive.

**Table:** Mumps virus neutralization test for serum of healthy human adults, Japan*

Serum sample no.	Patient age, y	Mumps history		EIA titer‡	NT titer†			
		Natural Infection	Vaccinated		rOdate	rOdate/ABMuV-F	rOdate/ABMuV-HN	rOdate/ABMuV-FHN
1	32	Unknown	+	2^2.35^	121	36	133	122
2	40	Unknown	–	2^1.36^	21	38	78	109
3	49	Unknown	–	2^2.09^	26	7	31	12
4	48	+	–	2^2.94^	176	122	183	149
5	58	+	–	2^2.80^	41	31	41	33
6	56	+	–	2^2.55^	31	8	85	27
7	40	Unknown	–	2^1.84^	88	105	58	33
8	35	+	–	2^3.32^	186	60	131	118
9	33	Unknown	–	2^2.48^	83	30	81	37
10	31	+	–	2^2.78^	53	5	122	26
11	18	Unknown	–	2^0.74^	<4	<4	<4	<4
12	18	Unknown	–	2^0.64^	<4	<4	<4	<4

*ABMuV, African bat mumps virus; EIA, enzyme immunoassay; F, fusion; HN, hemagglutinin-neuraminidase; NT, neutralizing; r, recombinant; +, positive; –, negative. †NT titer was determined as the dilution of serum that gave 50% plaque reduction compared with the average number of plaques formed in the absence of serum using the method of Reed and Muench. ‡Determined by using a commercially available indirect IgG eEIA kit (Mumps IgG-EIA kit; Denka Seiken Co., Niigata, Japan) according to the manufacturer’s instruction. Titers <2^1^ are seronegative, 2^1^–2^2^ are indeterminate, and >2^2^ are seropositive.

## Conclusions

To our knowledge, no infectious ABMuV has been isolated, although the entire genome sequence was detected in bats. To study the context of virus infection, we generated rMuVs carrying the ABMuV envelope proteins by reverse genetics. By using expression plasmids, Kruger et al. reported that the functions, such as fusion, hemadsorption, and neuraminidase activities, of the envelope proteins were conserved and compatible between MuV and ABMuV (*7*). These findings agreed with our data using the recombinant viruses, but notable differences existed. For example, Kruger et al. reported that the ABMuV envelope proteins induce smaller syncytia than the MuV proteins, whereas we observed enhanced syncytium formation by rMuV carrying the ABMuV HN protein. However, the enhancement was not due simply to the functional difference between MuV and ABMuV HN proteins because the HN proteins showed similar fusion-supporting capacities when expressed using expression plasmids. Further investigation of the involvement of other viral proteins modulating the HN protein function could lead to elucidation of the mechanism underlying this difference. Moreover, although Kruger et al. mentioned that the fusion activity might restrict the viral species specificity, our data indicated that the envelope proteins of MuV are not critical determinants of the host specificity in cultured cells. Our study also demonstrated that a synthetic genome strategy, which has also been used for the study of a bat influenza virus (*10*,*11*), is useful for the characterization and risk assessment of emerging viruses, even when the authentic viruses have not been isolated.

Our data showed extensive cross-neutralization between MuV and ABMuV. Although NT antibodies might play an essential role for MuV protection, a definitive NT titer for MuV protection is still under debate (*12*). Cortese et al. suggested that case-patients generally had lower preoutbreak mumps antibody levels than non–case-patients; however, no cutoff NT titer was defined in their study (*12*). Our findings suggest that antibodies induced by either mumps vaccines or infection with wild-type MuV generally neutralize ABMuV efficiently. Because cell-mediated immunity might also contribute to MuV protection (*13*), further investigations are needed to clarify the definitive parameters of MuV and ABMuV protection. Nonetheless, our data demonstrate that the current MuV vaccination program reduces the risk for an emerging infection of ABMuV in humans.

## References

[1] Drexler JF, Corman VM, Muller MA, Maganga GD, Vallo P, Binger T, Bats host major mammalian paramyxoviruses. Nat Commun. 2012;3:796. Correction in Nat Commun. 2014;5:3032. 10.1038/ncomms1796 PMC3343228 22531181

[2] Lamb RA, Parks GD. Paramyxoviridae: the viruses and their replication. In: Knipe DM, Howley PM, Griffin DE, Lamb RA, Martin MA, Roizman B, et al., editors. Fields virology. 5th ed. Philadelphia: Lippincott Williams & Wilkins; 2006. p. 1449–96.

[3] Hviid A, Rubin S, Muhlemann K. Mumps. Lancet. 2008;371:932–44 . 10.1016/S0140-6736(08)60419-5 18342688

[4] World Health Organization. Mumps virus nomenclature update: 2012. Wkly Epidemiol Rec. 2012;87:217–24 . 24340404

[5] Tsurudome M, Yamada A, Hishiyama M, Ito Y. Monoclonal antibodies against the glycoproteins of mumps virus: fusion inhibition by anti-HN monoclonal antibody. J Gen Virol. 1986;67:2259–65. 10.1099/0022-1317-67-10-2259 3760826

[6] Šantak M, Örvell C, Gulija TK. Identification of conformational neutralization sites on the fusion protein of mumps virus. J Gen Virol. 2015;96:982–90. 10.1099/vir.0.000059 25614584

[7] Krüger N, Hoffmann M, Drexler JF, Müller MA, Corman VM, Sauder C, Functional properties and genetic relatedness of the fusion and hemagglutinin-neuraminidase proteins of a mumps virus–like bat virus. J Virol. 2015;89:4539–48 and. 10.1128/JVI.03693-14 25741010 PMC4442385

[8] Saito H, Takahashi Y, Harata S, Tanaka K, Sano T, Suto T, Isolation and characterization of mumps virus strains in a mumps outbreak with a high incidence of aseptic meningitis. Microbiol Immunol. 1996;40:271–5. 10.1111/j.1348-0421.1996.tb03346.x 8709862

[9] Rubin S, Eckhaus M, Rennick LJ, Bamford CG, Duprex WP. Molecular biology, pathogenesis and pathology of mumps virus. J Pathol. 2015;235:242–52. 10.1002/path.4445 25229387 PMC4268314

[10] Juozapaitis M, Aguiar Moreira E, Mena I, Giese S, Riegger D, Pohlmann A, An infectious bat-derived chimeric influenza virus harbouring the entry machinery of an influenza A virus. Nat Commun. 2014;5:4448. 10.1038/ncomms5448 PMC5533278 25055345

[11] Zhou B, Ma J, Liu Q, Bawa B, Wang W, Shabman RS, Characterization of uncultivable bat influenza virus using a replicative synthetic virus. PLoS Pathog. 2014;10:e1004420. 25275541 10.1371/journal.ppat.1004420 PMC4183581

[12] Cortese MM, Barskey AE, Tegtmeier GE, Zhang C, Ngo L, Kyaw MH, Mumps antibody levels among students before a mumps outbreak: in search of a correlate of immunity. J Infect Dis. 2011;204:1413–22. 10.1093/infdis/jir526 21933874

[13] Vandermeulen C, Leroux-Roels G, Hoppenbrouwers K. Mumps outbreaks in highly vaccinated populations: What makes good even better? Hum Vaccin. 2009;5:494–6. 10.4161/hv.7943 19279405

